# Identifying longitudinal healthcare pathways and subsequent mortality for people living with dementia in England: an observational group-based trajectory analysis

**DOI:** 10.1186/s12877-024-04744-5

**Published:** 2024-02-14

**Authors:** James Watson, Mark A. Green, Clarissa Giebel, Asangaedem Akpan

**Affiliations:** 1 Department of Primary Care and Mental Health, The University of Liverpool, 1st Floor, Waterhouse Building B, Liverpool, L69 3GF UK; 2 School of Environmental Sciences, The University of Liverpool, Liverpool, UK; 3 Department of Primary Care and Mental Health, University of Liverpool, Liverpool, UK; 4 Applied Research Collaboration North West Coast, Liverpool, UK; 5 Department of Medicine for Older People and Stroke, Liverpool University Hospitals NHS FT, Liverpool, UK; 6 Healthy Ageing Group, University of Cumbria, Carlisle, UK; 7 Institute of Life Course and Medical Sciences, University of Liverpool, Liverpool, UK; 8 Clinical Research Network, North West Coast, Liverpool, UK

**Keywords:** Dementia, Healthcare, Pathway, Mortality, Trajectories, Temporal, Cluster, Inequalities

## Abstract

**Background:**

The number of people living with dementia (PLWD) continues to increase, particularly those with severe symptomatology. Severe symptoms and greater ill-health result in more acute care need. Early healthcare interventions can prove beneficial. Healthcare use has not been analysed as a holistic set of interlinked events. This study explores different healthcare pathways among PLWD, social or spatial inequalities in healthcare pathways and subsequent mortality risk.

**Methods:**

Group-based trajectory models (GBTM) were applied to electronic healthcare records. We generated clusters of PLWD with similar five-year, post-diagnosis trajectories in rates of primary and secondary healthcare use. Potential social and spatial variations in healthcare use clusters were examined. Cox Proportional Hazards used to explore variation in subsequent mortality risk between healthcare use clusters.

**Results:**

Four healthcare use clusters were identified in both early- (*n* = 3732) and late-onset (*n* = 6224) dementia populations. Healthcare use variations were noted; consistent or diminishing healthcare use was associated with lower subsequent mortality risk. Increasing healthcare use was associated with increased mortality risk. Descriptive analyses indicated social and spatial variation in healthcare use cluster membership.

**Conclusion:**

Healthcare pathways can help indicate changing need and variation in need, with differential patterns in initial healthcare use post-diagnosis, producing similar subsequent mortality risk. Care in dementia needs to be more accessible and appropriate, with care catered to specific and changing needs. Better continuity of care and greater awareness of dementia in primary can enhance prospects for PLWD. Research needs to further illuminate holistic care need for PLWD, including health and social care use, inequalities in care, health and outcomes.

**Supplementary Information:**

The online version contains supplementary material available at 10.1186/s12877-024-04744-5.

## Background

There are rising numbers of people living with dementia (PLWD) in the UK [1] with over 1 million projected by 2024. The greater proportional rise is set to be among those with severe dementia and more pressing health and care needs [2]. Such trends are placing increasing demands and costs on health and social care services [1]. The complex nature of care needs for PLWD contributes to the high costs of providing care [3, 4]. Understanding the different experiences of healthcare utilisation is therefore imperative if we are to align health systems to the care that PLWD need.

Good quality health and social care can support PLWD to live well and receive care at home longer [5, 6]. Living at home for longer is associated with improved physical health outcomes. quality of life [7] and lower mortality risk among PLWD [8–10]. Inadequate, ineffective or a lack of timely treatment can see rapid progression to more severe symptoms, requiring acute care sooner and more often [11–13]. PLWD are not only more likely to be admitted to hospital, but once they are, they are likely to stay longer in hospital and to be readmitted [14, 15]. Hospital stays can exacerbate dementia symptoms, impact physical health, and increase the likelihood of increased mortality [16, 17]. Issues with funding and service availability persist with many not being able to access timely diagnosis or appropriate treatment or support [18, 19].

There are wide social, demographic and geographical inequalities in the frequency and quality of healthcare received, quality of life and wellbeing, likelihood of transitions to care institutions, speed of dementia progression, severity of other chronic health conditions, and risk of mortality among PLWD [20–23]. It is a priority of the UK Government to address and reduce these inequalities [24]. A lack of central funding in the UK, including a legacy of austerity which saw cuts in funding that was greater in deprived areas, has limited the level and quality of care and treatment available [25]. These funding issues may disproportionally impact inequalities in access to health, and social, care, widening inequalities and resulting in poorer health and health outcomes for PLWD from disadvantaged backgrounds [4–26]. This illustrates the need to understand the differential experiences of healthcare utilisation among PLWD from different social and spatial groups. Currently, there is a lack of research exploring social and spatial determinants of healthcare use among PLWD resulting in a paucity of evidence on modifiable barriers to such inequalities.

Healthcare use is often analysed by focusing on one-off healthcare events or individual types of healthcare. However, this ignores the broader context of healthcare pathways [27, 28]. Healthcare pathways are a longitudinal sequence of linked contacts with healthcare services which can help demonstrate evolving needs and changing impacts on the health and health outcomes of an individual [29]. Health and social care have a cumulative impact on the health, survival, quality of life and health outcomes of PLWD [30]. Providing effective and good quality health and social care are vital to PLWD and their informal carers [31–33]. This is vital as needs for PLWD increase as their condition deteriorates [18]. It is beneficial to PLWD and their carers that they receive both pharmacological treatment and the variety of benefits which appropriate social care involvement can provide [34, 35]. Increased social isolation—as highlighted during the COVID-19 pandemic – increases the risk of rapid deterioration in memory and motor functions [26, 36]. Dementia can progress rapidly for some PLWD and symptoms of dementia and care need can change quickly and vary greatly over time, depending on dementia subtypes [18, 37]. Dementia subtypes can impact a person’s cognitive and motor functioning differently, which can in turn has a differential effect on somebody’s capability to manage finances [38].

This illustrates how vital the need for early, and correct, diagnosis and selection of appropriate health and social care provision is. It can help maintain independence and cognition for longer, delay more severe symptoms of dementia, manage other chronic conditions and improve survival among PLWD, as well as reducing the overall economic cost to the health and social care system [39–43]. There is a dearth of research which has investigated sequences of healthcare use for PLWD [21]. There is also a lack of studies investigating the simultaneous impact of multiple socio-economic, geographic and demographic factors in healthcare pathways and their resultant health outcomes [21]. Given healthcare use can play a critical role in future needs for care and health outcomes, it is vital to identify the different care pathways experienced by PLWD, and how these pathways can differentially impact health outcomes among PLWD.

Primary healthcare involvement is vital to treating dementia and other chronic conditions in PLWD and effective, consistent, holistic and person-centred primary healthcare can be central to a multifaceted support model which can help improve quality of life, maintain cognition and maintain care at home for longer, which can all enable better longer survival [44]. Levels of GP involvement and pharmacological treatment have been employed as outcomes measures in previous research [4, 21], and can indicate appropriateness of ongoing care for PLWD, and the degree to which medications prescribed are appropriate to the need of PLWD [45].

Three secondary healthcare use variables have been examined as outcome measures in previous research [21]: accident and emergency (A&E) attendances, emergency hospital admission spells and elective hospital admission spells. Acute hospital care, including admissions to hospital, is costly in terms of the health of the individual and financially to the healthcare system. Hospital admissions can often occur after changes in symptomatology and care needs [46], but can often be avoided through appropriate and effective care in the community [17]. PLWD are more likely to spend longer in hospital when admitted [47], to be readmitted to hospital [14], to move into a care home once discharged from hospital [48], and experience poor health outcomes following hospital admission [16, 17].

The aims of this novel data linkage study were to: (i) identify potentially different types of longitudinal trajectories of primary and secondary healthcare use among PLWD; (ii) examine how social and spatial inequalities persist across healthcare trajectory types; and (iii) analyse if different types of trajectories of healthcare are associated with different levels of survival in dementia.

## Methods

### Data access and ethical approval

We used pseudonymised routinely collected Electronic Health Records (EHR) from Clinical Practice Research Datalink (CPRD) Aurum [49]. CPRD contains data for 18 million currently active patients registered with UK General Practices (GP). CPRD includes patient details and demographics, primary (GP observations and medication prescriptions) and linked secondary healthcare contacts (Accident and Emergency (A&E) attendances and hospital admission spells). Access to data for the purposes of specified research was granted by CPRD and ethical approval for the use of CPRD Aurum was provided by the University of Liverpool Research Ethics Board (Reference: 7922).

### Loss-to follow-up and missing data

Loss to follow-up could occur through an individual dying or having changed to a GP who was not registered with CPRD. If a member of the sample population was lost to follow-up during a specific year after diagnosis, we gave the number of healthcare contacts (of all four types) in the years following loss to follow-up as “NA”, as they were no longer present in the data (censored).

Some people will have been present during a specific year after diagnosis, or throughout the time period, but did not have recorded contact(s) with one or more of the healthcare service types. In this case, they were given a value of 0 contacts for that healthcare service type(s). In this study, loss to follow-up increased beyond the populations’ 5th year post-diagnosis. As such, only people remaining in the study five years after diagnosis (5 years of complete data post-diagnosis) data were included in statistical analysis, including GBTM and subsequent cluster-survival analysis.

The original sample population for those living with early- and late-onset dementia were 5,210 and 137,077 respectively. Some of the sample population had fewer than five years of post-diagnosis healthcare contact data and were therefore defined as lost to follow-up (Additional file 1). From those original sample populations, almost three quarters of those with early-onset (3,735; 71.7%) and less than half of those with late-onset (62,264; 45.5%) dementia were included in GBTM. Details of the final sample population included in the GBTM and subsequent analyses, from both early- and late-onset populations, are detailed in the following section.

### Sample population

Our sample population contains people registered with a CPRD-registered General Practice who received a diagnosis of dementia between the years 2002 and 2016. Dementia diagnosis in this case refers to patients on GP registers who have been diagnosed with a condition related to one or more of the read codes associated with dementia (Additional file 6). Following application of the inclusion criteria (defined in previous section), the final analytical sample size for early-onset was 3735, and for late-onset dementia was 62 264. We stratified our sample population by dementia-onset, with early-onset (aged < 65 years) and late-onset dementia (aged 65 +) split into concurrent analyses.

### Outcome variable

Mortality was our outcome based on the presence of a date of death in CPRD. Mortality within our population could occur between the 1st and 14th year after the five-year trajectory of healthcare use.

### Healthcare use trajectories

Healthcare pathways are made up of multiple strands of unique healthcare service types. Here we have included four types of healthcare as trajectories for each member of the sample population:

1. *GP observations* are single records of each observation at a GP visit. Multiple observations can occur at a patient-GP consultation, with each observation related to a different matter discussed.
2. *Dementia medication prescriptions* relate to four NHS-advised drugs for treatment of dementia: Donepezil, Galantamine, Rivastigmine and Memantine (extracted based on aforementioned ‘Product Names’ from ‘Drug_Issue’ files within the CPRD data).
3. *Non-dementia medications prescriptions*: refer to all other medications than the four NHS-advised medications for the treatment of dementia (all other medications from ‘Drug_Issue’ files within CPRD data).
4. *Acute secondary healthcare* includes combined records for:
  - Accident & Emergency attendances: unplanned presentations at A&E or urgent care.
  - Hospital admission spells: patient requires further treatment or observation.

Records in which an A&E attendance may have led to a hospital admission, these are counted as separate records, and counted as such in analyses. Each of the four healthcare use variables were calculated initially as counts in each calendar year, for each person. Within group-based trajectory models, the values for each of the four healthcare types, across the five-year period, is based on the z-score for the cluster (standardised to the mean for the cluster).

### Temporal healthcare use

Year of diagnosis was used as year 0 and only healthcare contacts occurring in the same calendar year are included in year 0. As such, if somebody was diagnosed later in the year, the potential for healthcare contacts was reduced compared to people diagnosed earlier in the year. Due to this potential issue, we have therefore removed year 0 healthcare contacts from any analyses, and instead healthcare contact data begins at year 1 – the first full, potential year of data for each member of the sample population. Calendar year was used for all temporal-based calculations, as the original CPRD data only included year for some temporal variables. Specifically, year of birth, which was used for calculating participant age, and the stratified dementia-onset category, led to only year-based date variables being used across the study.

Attrition and years of survival beyond dementia diagnosis meant it was necessary to define a time period from which the analysis would be based. To maintain integrity in the study and validity of findings we restricted healthcare records to those which occurred between the first and fifth years of post-diagnosis healthcare records. This falls in-line with dementia survival estimates. It was also pragmatic to negate the potential impact of attrition and to attain a substantial temporal trajectory of healthcare use among a representative population sub-sample. At the five-year point loss to follow-up was ~ 79% in early-onset and ~ 58% for late-onset sample populations.

### Explanatory factors

This study looks to describe each of the aforementioned clusters derived from GBTM, based on their composition. Identification of the socio-economic, demographic and geographic make-up of each of the clusters derived for both early- and late-onset dementia.

Previous research has identified multiple potential explanatory factors of variation in healthcare use and health outcomes for PLWD. Studies have explored a range of potential explanatory factors for differential healthcare use and mortality risk inequalities. CPRD data and data linkage provides patients’: age at diagnosis, sex, ethnicity, 2015 Indices of Multiple Deprivation (IMD) quintile and GP urban/rural classification and GP region. Research has shown how variations in healthcare utilisation and health outcomes for PLWD vary across these key factors [21–23, 50].

### Statistical analysis

All statistical analyses, including descriptive statistics, data visualisation, regression analyses and group-based trajectory modelling was conducted in rStudio [51]. Initial descriptive analysis demonstrates the demographic, socio-economic and geographic composition of the stratified sample populations. Clusters from GBTM receive a probability value for each member of the cluster having been correctly assigned. Each sample population member receives a value indicating the likelihood of belonging to each of the clusters generated, having been assigned to the cluster they’re deemed most likely to belong [52].

GBTM as a statistical method allows for a sample population to be grouped based on similarities in temporal changes across multiple measures [53]. In this case we have employed GBTM to generate groups of PLWD based on similar patterns in their use of GP observations, dementia medications, non-dementia medication and acute secondary healthcare. GBTM is a data driven approach where the number of groups needs to be specified a priori.

To identify the best fitting number of groups, we ran the model for between one and ten cluster groups. We select up to 10 groups since we want to a parsimonious model that maximises variability across groups, but also minimises the complexity that each additional group brings. Model fit was then compared using Bayesian Information Criterion (BIC) and Log-likelihood (logLik) (Additional file 2), with visual trajectory plots for healthcare use trajectories for each number of cluster (k) used to aid in the number of final clusters used for mortality risk analyses. The restrictive level of computing power needed to run the models on such a large number of data points across a large population meant it was not practical to do so. Instead a 10% sample of the overall late-onset population was extracted to for GBTM, with a second 10% sample population also taken to validate and ratify the original GBTM and subsequent outputs.

Descriptive statistics of the social and spatial composition, and subsequent mortality for each cluster were calculated. Demographic, spatial and socio-economic differences in cluster membership was analysed using multinomial logistic regression. Analysis of mortality risk across each healthcare trajectory clusters was performed using Cox Proportional Hazards regression.

Survival was analysed for up to 14 years beyond the healthcare use trajectory period. In survival and mortality analysis, it is possible for data to be right-censored. That is, they leave the study before they may encounter the event of interest (mortality). In this study, it is possible that, given the long follow-up period of 14 years beyond the initial five-year healthcare trajectory period, that members of the sample population did not die, but they were lost to follow-up. This can be because they withdrew their consent for their GP to send their data to CPRD, or that they changed GP, from one which was initially registered with CPRD, to one which was not, and as such their data was no longer sent to CPRD.

The potential issue of right censoring was addressed through analyses. Mortality risk was analysed using Cox Proportional Hazards regression and Kaplan–Meier curves, which only include the sample population as ‘at-risk’ of the outcome if they remain in the data. They are removed from the analyses at the point at which their data ends (e.g. if they died, did not have the event of interest, or, did not have any subsequent data).

All regression models, including testing for associations between: (1) healthcare use cluster membership and socio-demographic and geographic factors, and (2) for cluster membership and mortality risk and survival adjusted for multiple potentially confounding factors: age at diagnosis, sex, ethnicity, IMD 2015 quintile, urban–rural GP classification and GP region as potential confounders.

## Results

### Sample population characteristics

Within our early-onset sample population there were 3,732 people. The majority were female (2,027; 54.3%), aged 55–64 (3,061; 82.0%) and registered with urban GP (3,234; 86.7%). The majority were from White ethnicity groups (3,267; 87.5%), with Asian (95; 2.5%), Black (88; 2.4%) and Mixed/Other (40; 1.1%) ethnicity groups making up much smaller proportions of the early-onset population. There were more people registered with GPs in certain regions of the country, including the North West (763; 20.4%), South Central (516; 13.8%) and West Midlands (617; 16.5%). The population was relatively evenly spread across areas of deprivation, with 724 (19.4%) in the most deprived quintile and 683 (18.3%) in the least deprived quintile.

There were 6,224 people in the late-onset GBTM population. The majority were female (68.9%), aged 75.84 (53.1%), registered with urban GPs (85.8%). It should be noted that in the late-onset population there was more missing data for ethnicity, however the late-onset population less ethnically diverse than the early-onset, with 1.3%, 1.9% and 0.9% from Asian, Black and Mixed/Other ethnicity groups respectively. More of the late-onset population lived in areas of less deprivation, with the least and second least deprived quintiles making up a combined 47.0%. As with early-onset, some GP regions made up a much greater proportion of the population; the North West (1,178; 18.9%), South Central (843; 13.5%), South West (883; 14.2%) and West Midlands (1,040; 16.7%).

### Attrition from sample population

We also found evidence of inequalities in attrition patterns, which may impact how generalisable our sample population is (Additional file 3). In early-onset dementia loss to follow-up among men and those aged 45–54 greater than their counterparts. Men, older people (aged 85–94 and 95 + years) and those from White ethnicity groups also had greater attrition than their counterparts.

### Sample selection

There was loss to follow-up from our sample population. From the original sample of 5,210 and 137,092 people with early- and late-onset dementia respectively 3,732 (71.6%) and 62,244 (45.4%) remained once we filtered for only those with at least five years of post-diagnosis healthcare records within our dataset. With a long observation period for the event of interest (mortality) there was further loss to follow-up. A 10% sample of our overall 62,244 late-onset population were included in GBTM models. From the 3,732 early- and 6,224 late-onset populations included in GBTM models, 1,126 (30.2%) and 2,548 (40.9%) had a date of death stated. Of the remaining 2,606 early-onset and 3,676 late-onset who had not died during the follow-up study period (in the 14 years following the five-year healthcare use trajectory period, which was included in subsequent mortality and survival analysis), nearly all did not have healthcare records for the entire study period; 2,595 (99.6%) early-onset and 3674 (~ 100%) late-onset. Data for these individuals was censored at the year of their final healthcare record(s). (Additional file 7).

### Selection of healthcare use trajectory clusters

**Fig. 1:**
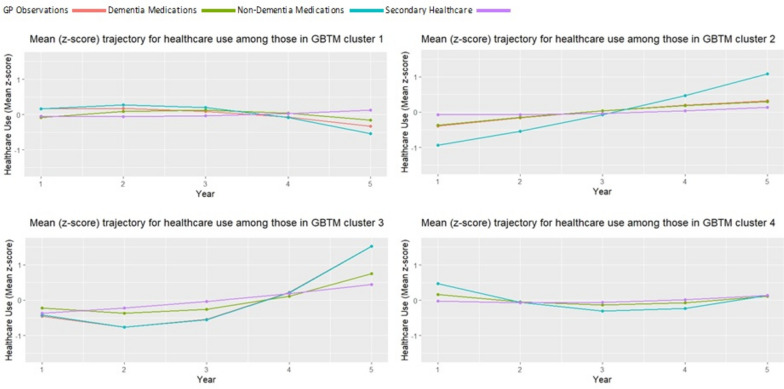
Early-onset sample population: Trajectories for mean use of each healthcare types in each group-based trajectory model (GBTM) derived cluster. Trend in the z-score (value in relation to the mean) for GP observations (red line), dementia medications (green line)), non-dementia medications (blue line) and secondary healthcare contacts (purple line) foreach healthcare use cluster within the sample population with early-onset dementia, across the first, full five years of healthcare contact data post-dementia diagnosis

**Fig. 2:**
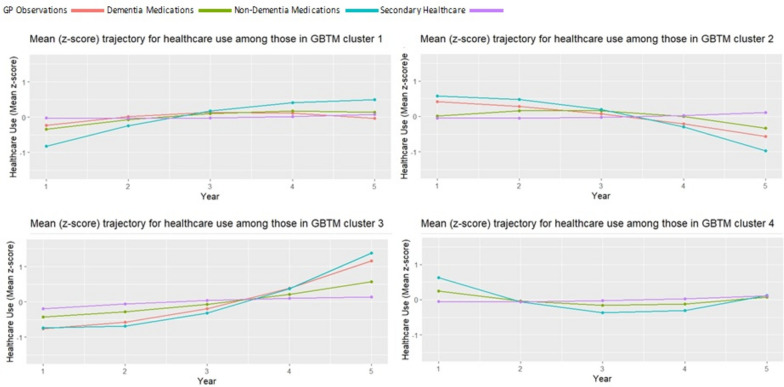
Late-onset sample population: Trajectories for mean use of each healthcare type in each group-based trajectory model (GBTM) derived cluster. Trend in the z-score (value in relation to the mean) for GP observations (red line), dementia medications (green line)), non-dementia medications (blue line) and secondary healthcare contacts (purple line) foreach healthcare use cluster within the sample population with late-onset dementia, across the first, full five years of healthcare contact data post-dementia diagnosis

The selection of number of groups was data driven. Our goal was to maximise information captured by having additional groups, while minimising the complexity of more groups. For both early and late-onset populations, four-group solutions were selected as the parsimonious solution (Additional file 2). Four groups were selected following comparing model fit, since additional groups only produced incremental model fit improvements (i.e., four groups was the elbow point). In addition, the visual representation of the healthcare trajectories (Figs. 1 and 2) for models including five or more groups did not incorporate any significant, additional experience in healthcare use trajectories.

### Defining healthcare use trajectory clusters

#### Early-onset

We found the following five-year post-diagnosis healthcare trajectory groups for people living with early-onset dementia (Fig. 1):

- *Group 1: ‘Drop-off in medicative treatment’* was comprised of 54.0% of those with early-onset*.* With the lowest rates of GP observations and medications at the end of the trajectory period, this group is characterised by slight reductions in GP contact and medications over the five years (trends are flat up to year 3 prior to declining).
- *Group 2: ‘Growing treatment of other chronic conditions’* contained 37.6% of those with early-onset dementia. Group 1 was characterised by larger year-on-year increases in prescriptions for non-dementia health conditions, as well as smaller annual increases in GP observations and dementia medications.
- *Group 3: ‘Late increases in healthcare use’* contains 5.1% of people with early-onset dementia. For the initial three years, the group has below average values for all measures. This is then followed by exponential increases in GP contacts and non-dementia medications (and to a lesser extent dementia medications). This group had marginal increases in secondary healthcare use.
- *Group 4: ‘Stable GP contact*’ contained 3.3% of those with early-onset dementia. With the highest rate of all primary healthcare contacts at the start of the period, this group is characterised by falling rates into year three where measures level off and then increase in year five.

#### Late-onset

We found the following five-year post-diagnosis healthcare trajectory groups for people living with late-onset dementia (Fig. 2):

- *Group 1: ‘Steady primary care involvement’* contained 44.2% of the late-onset sample population. Group 3 was characterised by small and consistent increases in each healthcare measure up to year 3 where the trend starts to level off.
- *Group 2: ‘Falling medicative treatments’* contained 38.9% of those living with late-onset dementia. This group was typified by reductions in primary healthcare and both types of medications over the five-year period.
- *Group 3: ‘Exponential increases in GP contact and medications’* contained 10.5% of those living with late-onset. This group was characterised by exponential increases in GP involvement and medications. By year five, the group has the highest values across all four measures of any cluster.
- *Group 4: ‘Heightened initial primary care, then steady GP involvement’* contained 6.4% of the population living with late-onset dementia. This group was defined by initial high values across measures in year 1, followed by declining values over time that see it with the lowest values by year 3. In years 4 and 5, trends reverse and measures begin to increase.

### Social and spatial variations in cluster membership

Descriptive and regression analysis highlighted differences in the demographic, geographic and socio-economic makeup of early- and late-onset clusters derived from GBTM (Table 1). Multinomial logistic regression also highlighted these variations in cluster membership (Additional file 4).

**Table 1:** % representation of early- and late-onset sample populations in each cluster, by demographic, geographic and socio-economic variables

Social/Spatial Factor	Cluster % representation: early-onset population				Total EoD		Cluster % representation: late-onset population				Total LoD	
	Cluster 1	Cluster 2	Cluster 3	Cluster 4	n	%	Cluster 1	Cluster 2	Cluster 3	Cluster 4	n	%
*Age Group*	*n* = *189*	*n* = *125*	*n* = *2016*	*n* = *1402*			*n* = *651*	*n* = *2422*	*n* = *401*	*n* = *2750*		
Under45	1.6%	4.8%	1.9%	2.1%	77	2.1%	*Not Applicable*					
45–54	15.3%	12.8%	16.5%	15.4%	594	15.9%	*Not Applicable*					
55–64	83.1%	82.4%	81.5%	82.5%	3061	82.0%	*Not Applicable*					
65–74	*Not Applicable*						21.7%	19.2%	15.2%	23.6%	1316	21.1%
75–84	*Not Applicable*						56.2%	52.3%	49.1%	53.7%	3306	53.1%
85–94	*Not Applicable*						21.8%	27.6%	34.7%	22.0%	1554	25.0%
95 +	*Not Applicable*						0.3%	0.9%	1.0%	0.7%	48	0.8%
*Sex*												
Female	49.2%	58.4%	56.6%	55.4%	2027	54.3%	69.6%	68.5%	73.1%	68.5%	4289	68.9%
Male	50.8%	41.6%	43.4%	44.6%	1705	45.7%	30.4%	31.5%	26.9%	31.5%	1935	31.1%
*Ethnicity*												
Asian	1.1%	0.0%	2.8%	3.0%	95	2.7%	0.7%	1.6%	0.8%	1.4%	80	1.3%
Black	2.2%	3.5%	2.7%	2.2%	88	2.5%	1.3%	1.8%	1.0%	2.4%	117	2.0%
Mixed/Other	2.2%	1.7%	1.3%	0.7%	40	1.1%	0.5%	1.3%	0.8%	0.7%	54	0.9%
White	94.4%	94.8%	93.1%	94.1%	3267	93.6%	97.6%	95.3%	97.4%	95.5%	5676	95.8%
*IMD 2015 Deprivation Quintile*												
5 Least Deprived	21.3%	12.8%	18.4%	18.4%	683	18.4%	20.9%	24.0%	26.3%	24.5%	1493	24.0%
4	23.4%	25.6%	21.9%	22.9%	837	22.5%	23.2%	24.0%	22.8%	22.2%	1432	23.1%
3	19.1%	22.4%	20.7%	20.8%	771	20.7%	16.9%	18.3%	18.5%	20.9%	1200	19.3%
2	18.1%	20.0%	18.9%	18.9%	703	18.9%	19.4%	18.8%	17.5%	17.4%	1129	18.2%
1 Most Deprived	18.1%	19.2%	20.0%	18.9%	724	19.5%	19.5%	14.9%	15.0%	14.9%	958	15.4%
*Urban–Rural GP Classification*												
Rural	14.8%	16.8%	12.2%	14.5%	498	13.3%	12.0%	14.7%	14.7%	14.1%	882	14.2%
Urban	85.2%	83.2%	87.8%	85.5%	3234	86.7%	88.0%	85.3%	85.3%	85.9%	5342	85.8%
*GP Region*												
East Midlands	2.6%	2.4%	2.7%	3.4%	110	2.9%	1.4%	2.6%	2.5%	2.1%	141	2.3%
East of England	3.2%	4.8%	5.4%	4.9%	189	5.1%	5.1%	5.2%	4.5%	5.7%	335	5.4%
London	10.1%	18.4%	13.1%	10.4%	453	12.1%	10.9%	10.7%	12.7%	10.5%	668	10.7%
North East	3.7%	2.4%	4.9%	5.7%	189	5.1%	6.5%	5.3%	2.5%	6.2%	351	5.6%
North West	24.3%	16.8%	20.5%	20.1%	763	20.4%	18.4%	19.4%	20.2%	18.4%	1178	18.9%
South Central	16.4%	18.4%	12.3%	15.3%	516	13.8%	15.1%	12.8%	14.5%	13.7%	843	13.5%
South East Coast	7.9%	12.8%	8.2%	6.9%	294	7.9%	8.6%	8.5%	10.5%	7.9%	520	8.4%
South West	11.6%	10.4%	11.9%	12.3%	447	12.0%	16.1%	13.0%	12.0%	15.1%	883	14.2%
West Midlands	15.3%	10.4%	16.7%	17.0%	617	16.5%	14.4%	18.2%	16.5%	16.0%	1040	16.7%
Yorkshire & The Humber	4.8%	3.2%	4.3%	3.9%	154	4.1%	3.5%	4.3%	4.2%	4.4%	265	4.3%

Proportion representation among each healthcare use cluster, among both early- and late-onset dementia sample populations, by socio-economic and geographic factor, in relation to the overall proportion of representation among the entire sub-sample population (either those with early- or late-onset dementia). Table demonstrates the differential membership of healthcare use clusters, for both early- and late-onset dementia sample populations, based on socio-economic and geographic characteristics

#### Characteristics of early-onset healthcare trajectory clusters

Compared to the overall breakdown of the early-onset population (female = 54.3%, male = 45.7%), there was a greater proportion of women in the *Stable GP contact* cluster (58.4%) and men in the *Late increases in healthcare use* cluster (50.8%). Compared to the make-up of the overall population by age, a greater proportion of those aged under 45 in the *Stable GP contact* cluster (4.8%). The least deprived and second least deprived IMD quintiles were more greatly represented in the *Late increases in healthcare use* (21.3%) and *Stable GP contact* (25.6%) cluster respectively and those registered with rural GPs made up a higher proportion of those in the *Stable GP contact* cluster (16.8%). Differences in the make-up of clusters were also seen by GP region: the London and South-East Coast regions were overrepresented in the *Stable GP contact* cluster, the North-West in the *Late increases in healthcare use* cluster and the South-Central region in both *Late increases in healthcare use* and *Stable GP contact* clusters. Multinomial logistic regression was conducted to highlight significant differences in the social and spatial breakdown of healthcare use cluster populations, with *Drop-off in medicative treatment* as our reference cluster. Though descriptive statistics indicate numerous variations in the breakdown of different clusters, few significant differences were found and only in the *Stable GP contact* cluster: aged under 45 (RR: 1.05; CI: 0.14–1.95), from deprivation Quintiles 1 (RR: 0.83; CI: 0.11–1.55) and 2 (RR: 0.72; CI: 0.03–1.41), and in London (RR: 1.76; CI: 0.27–3.26), South Central (RR: 1.77; CI: 0.26–327) and South East Coast (RR: 1.65; CI: 0.12–3.18) GP regions.

#### Characteristics of late-onset healthcare trajectory clusters

A greater proportion of the late-onset population were women (68.9%) compared to 31.1% men. Women were even more greatly represented in the *Heightened initial primary care, then steady GP involvement* cluster (73.1%). Differences in representation were also evident based on age group: those aged 65–74 were overrepresented in the *Steady primary care involvement cluster* (23.8%), those aged 75–84-year olds in the *Exponential increases in GP contact and medications* cluster (56.4%) and those aged 85–94-year olds in the *Heightened initial primary care, then steady GP involvement* (35.0%) and *Falling medicative treatments* (27.9%) clusters. The least deprived IMD quintile was overrepresented in the *Heightened initial primary care, then steady GP involvement* cluster (26.3%) and the most deprived in the *Exponential increases in GP contact and medications’* (19.5%), and those with urban GPs more greatly represented in the *Exponential increases in GP contact and medications’* (88.0%). The South-East Coast GP region was overrepresented in the *Heightened initial primary care, then steady GP involvement* cluster (10.5%). Multinomial logistic regression found few significant differences in the social and spatial breakdown of late-onset dementia healthcare use clusters. Compared to *Steady primary care involvement*, all clusters had more people aged 75–84 and 85–94 and some variation by GP region. The *Exponential increases in GP contact and medications* cluster also had significantly more from deprivation Quintile 1 (RR: 0.49; CI: 0.19–0.78) and fewer from Black ethnicity groups (RR: (-)0.86; CI: (-)1.62-(-)0.10).

## Please place Table 1 here (originally placed landscape due to dimensions of the table)

### Healthcare use cluster survival

#### Early-onset

**Fig. 3:**
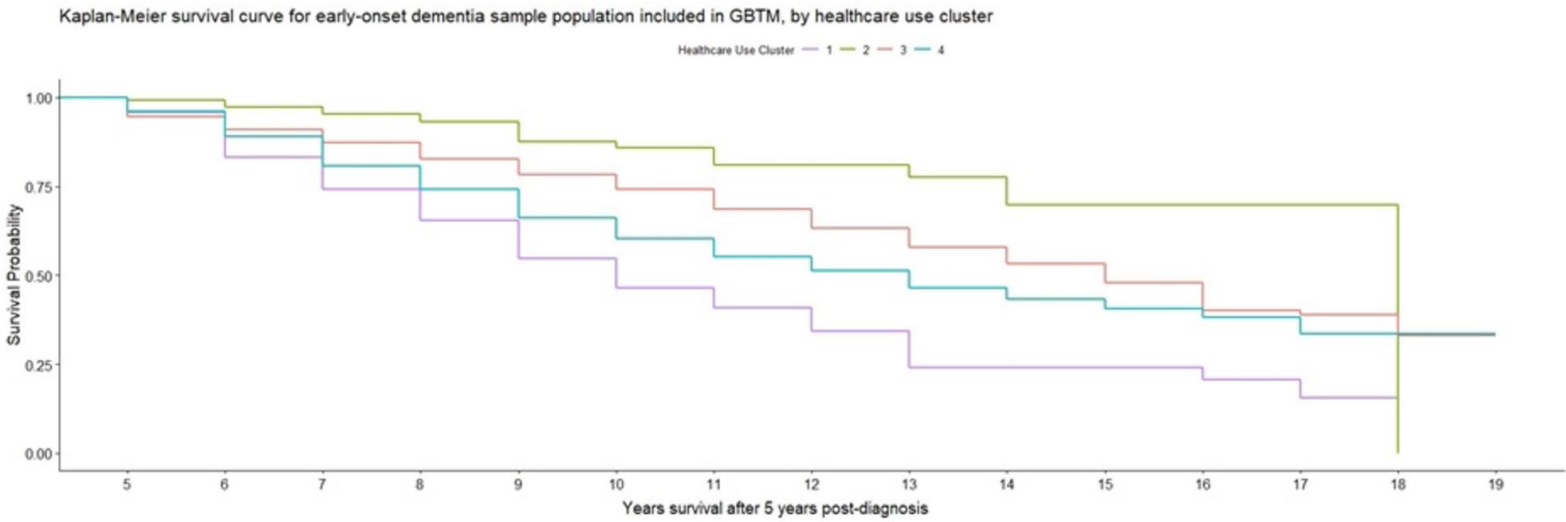
Kaplan–Meier survival curve for sample population with early-onset dementia included in GBTM, by healthcare trajectory cluster. Time-to-event analysis (% survival or loss to follow-up) for people in early-onset dementia sample sub-population, between years 5 to 19 after their dementia diagnosis, based on healthcare use clusters derived from group-based trajectory models for healthcare use in the five years after their dementia diagnosis. Healthcare use clusters 1 (purple line), 2 (green line), 3 (red line) and 4 (blue line) display differential rates of survival/loss to follow-up over the period analysed in time-to-event analysis

Our final analyses used cox regression models to examine if there were statistically significant differences in survival between the four clusters. In the early-onset sample population, compared to our reference cluster (*Drop-off in medicative treatment*), the cluster *Stable GP contact* had a significantly lower risk of mortality (HR: 0.47; CI: 0.28–0.77), whereas both *Growing treatment of other chronic conditions’* (Hazard Ratio (HR): 1.37; Confidence Intervals (CI): 1.21–1.56) and *Late increases in healthcare use* (HR: 2.21; CI: 1.78–2.75) had significantly greater risk of subsequent mortality beyond the five-year healthcare trajectory period (Additional file 5). Kaplan–Meier survival curves (Fig. 3) also graphically demonstrate the poorer survival among those in the *Growing treatment of other chronic conditions* and *Late increases in healthcare use* clusters. A larger percentage of people in *Growing treatment of other chronic conditions’* (22.9%) and *Late increases in healthcare use* (32.8%) had died within three years of the end of our trajectories, compared to lower rates of mortality in clusters *Stable GP contact* (5.6%) and *Drop-off in medicative treatment* (13.6%).

The clusters with the greatest mortality risk—*Growing treatment of other chronic conditions’* and *Late increases in healthcare use*—both had healthcare trajectories defined by initial lower than average rate of GP observations and prescriptions for both dementia and non-dementia medications, followed by increases in values over time that saw them have the highest values and use of healthcare. The magnitude of the differences in the effect sizes modelled may also reflect the differences in the trajectory, with *Late increases in healthcare use* having a steeper and larger rise in healthcare utilisation and also a larger hazards ratio. *Stable GP contact*, which had a significantly lower risk of mortality than our reference cluster (*Drop-off in medicative treatment*), had more settled rates of GP contacts and medications.

#### Late-onset

**Fig. 4:**
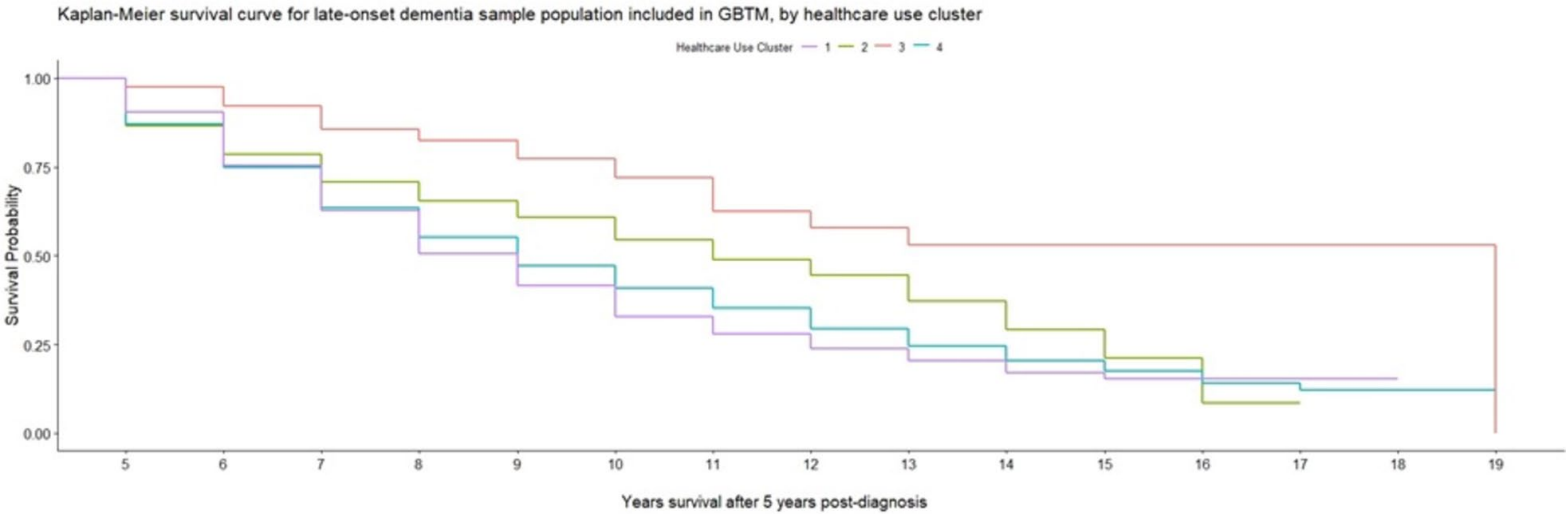
Kaplan–Meier survival curve for late-onset GBTM population, by healthcare trajectory cluster. Time-to-event analysis (% survival or loss to follow-up) for people in late-onset dementia sample sub-population, between years 5 to 19 after their dementia diagnosis, based on healthcare use clusters derived from group-based trajectory models for healthcare use in the five years after their dementia diagnosis. The different healthcare use clusters 1 (purple line), 2 (green line), 3 (red line) and 4 (blue line) experience variations in their rates of survival/loss to follow-up over the period analysed in time-to-event analysis

We repeated our cox regression analyses for people living with late-onset dementia (Additional file 5). With the cluster *Steady primary care involvement* as the reference group and accounting for all socio-economic, demographic and geographic factors as confounders, we found that mortality risk was significantly lower in cluster *Heightened initial primary care, then steady GP involvement* (HR: 0.35; CI: 0.25–0.40) and *Falling medicative treatments* (HR: 0.72; CIs: 0.66–0.80). This is further illustrated by Kaplan–Meier survival curves demonstrating increased survival in these clusters (Fig. 4). Both the *Heightened initial primary care, then steady GP involvement* and *Falling medicative treatments* – those with significantly lower mortality risk than our reference cluster – had declining trends in healthcare utilisation over time. No statistically significant difference was found for *Exponential increases in GP contact and medications* compared to *the Steady primary care involvement cluster*. However, there are potential issues and biases resulting from attrition rates in this study, particularly among the late-onset dementia population study. With less than 50% of the initial late-onset sample population included in temporal group-based trajectory models, and the healthcare period covering five years post-diagnosis, the findings presented (healthcare use and survival) may not be entirely representative of the late-onset population as a whole, or of the socio-demographic groups identified.

### Early-onset

Figure 3.

### Late-onset

Figure 4.

## Discussion

This study is one of the first to employ large-scale electronic health records to define clusters of PLWD in their use of primary and secondary healthcare use to demonstrate the different pathways PLWD encounter in the years beyond their diagnosis. We also demonstrate how these different healthcare trajectories vary across social and spatial inequalities, as well as how these patterns translate to mortality risk. In people living with late-onset dementia, we defined four groups including ‘*Heightened initial primary care, then steady GP involvement*’ and ‘*Falling medicative treatments*’. The former saw changes over the five-years in primary healthcare use. High initial rates were followed by a reduction and subsequent late rise in primary healthcare use. The latter witnessed consistent reductions in primary healthcare use and medications. Both clusters had significantly lower mortality risk than our reference cluster ‘*getting to grips with treatment*’ (a cluster defined by lower uptake of healthcare). Among people with early-onset dementia, we also defined four groups. The ‘*Growing treatment of other chronic conditions’*’ cluster had increases over the period in all three primary healthcare variables, ‘*Late increases in healthcare use*’ showed low healthcare use initially, followed by late, exponential increases in healthcare use and, ‘*Stable GP contact*’ at the end of the five years, had the lowest rates of GP contact and medications. Differential mortality risk was noted between these clusters which did not seem to be specific to one particular type of healthcare use trajectory. Compared to our reference cluster (‘*Drop-off in medicative treatment*’) higher mortality risk was observed in both ‘*Growing treatment of other chronic conditions’*’ and ‘*Late increases in healthcare use*’ and lower mortality risk was observed in ‘*Stable GP contact*’.

Through GBTM, we demonstrate that in the years following a dementia diagnosis, PLWD can experience differential levels of contact with primary healthcare, medications and secondary healthcare use. PLWD have greater, and more severe, other chronic conditions than the general population [54, 55]. Additional chronic health conditions and the complexity of treating dementia can result in increased need for a greater range of healthcare among PLWD [56]. However, care need can be complex and unique for PLWD [57] and as dementia progresses it can quickly alter what a PLWD requires [58]. Our findings show that this complexity in need could potentially produce different types of healthcare experiences that do not necessarily correspond to increasing need over time. Increased contact between a PLWD and their GP may be beneficial [8]. However, increased GP contact and medications may be a result of polypharmacy resulting from a lack of appropriate medication reviews or care management [59]. Therefore, clinicians need to discuss with PLWD and carers the intended purpose and potential impacts of medications to make informed decisions on their use [60]. While no two PLWD are the same and their experiences will depend on their specific needs [61], there are collective similarities in experiences of healthcare [62, 63].

Our study also demonstrates that for both early- and late-onset dementia, different trajectories of healthcare use were associated with different subsequent mortality risks. In both early- and late-onset dementia exponential increases over the trajectory resulted in higher mortality risk. This study also highlights that consistent, or slowly diminishing rates of primary healthcare contact were associated with lower mortality risk. This would seem to indicate that PLWD who are receive appropriate treatment and care management from diagnosis experience longer-term health benefits [64, 65]. Those who may not receive effective treatment early-on may endure poorer quality care as time goes on – in the form of increased inappropriate medications, which can result in poorer health outcomes [64]. These trajectories may emphasise the importance of acting early and appropriately in providing healthcare [41, 66]. Good primary healthcare in dementia does not necessarily mean increased service involvement, but rather that services need to be aware of changing needs for PLWD and be on-hand to provide timely and effective care [58]. Meeting specific and changing needs of PLWD is essential to providing the quality and consistency of care required to allow better quality of life and reduce mortality risk [8, 67]. The different clusters identified may potentially indicate the potential benefits of tailored care, identifying need and future risks as a better means of managing care. Understanding patient pathways through the health system, including matching people to their most appropriate pathway, may help to improve health outcomes among PLWD. This is because PLWD are also more likely to experience ineffective or inappropriate healthcare use, including inappropriate medications [64], unnecessary transitions into nursing care [68] and avoidable emergency healthcare use [69]. Ineffective healthcare use is associated with increased negative health outcomes [70] and greater financial cost to health and social care services [13, 71].

In addition to our findings related to healthcare use pathways and subsequent morality risk, our study highlights some social and spatial groups of PLWD are more likely to go through certain healthcare pathways, and may therefore be at greater risk of differential health outcomes including mortality risk. Our healthcare trajectories highlight how PLWD from deprived or urban areas were more likely to belong to clusters associated with inadequate need or delayed care access. Receiving inappropriate treatment, encountering issues with service equity and accessibility and, poor care quality is more likely among PLWD from ethnic minority backgrounds [21, 72, 73], more deprived [20, 21, 25] and rural areas. As these groups are more greatly impacted by unmet care needs [62, 74], they are at greater risk of negative care and the associated poor health outcomes, including lower quality of life, and increased falls risk, emergency healthcare use [14] and mortality risk [62, 75, 76]. The causes of healthcare trajectory variations by different social and spatial groups of PLWD are nuanced. Differences in geographic provision and local service finances [77, 78], variation in accessibility and appropriateness for different population groups, and disparity in the quality of care and support [21] meaning PLWD encounter contrasting care pathways which impact the likelihood of poor health outcomes. However, the complex inequalities in healthcare trajectories we note, combined with associated differential mortality risk, may contribute to explaining social and spatial inequalities in dementia outcomes.

### Limitations

Loss to follow-up and attrition have been discussed previously, and we highlight again that a substantial proportion of our original early- and late-onset sample populations were not included in our analyses. Research suggests that loss to follow-up of less than 5% of the sample population is unlikely to lead to any bias, but greater attrition will begin to impact validity of findings at 20% [79, 80]. There is the potential for attrition bias in such research, with members of some demographic groups being lost to follow-up earlier than others. The overall loss to follow-up rate by year five of the healthcare trajectory was greater than the level at which bias can be introduced (20%), for both early- and late-onset sub-sample populations. Although the CPRD sample is approximately 25% of the UK’s GP patient population, and is representative of the overall UK population, if a GP opts out of CPRD or a patient leaves a CPRD practice for a non-CPRD GP, their data will end at this point. The loss to follow-up experienced in this study may have introduced selection bias in our sample population. Loss to follow-up, and exclusion of people with less than five years of healthcare use data available post-dementia diagnosis may be more likely among groups who are more likely to experience delays or incorrect diagnoses [81, 82]. These groups include people from ethnic minority backgrounds and from more socio-economically deprived areas, meaning the findings and narrative discussed may not be entirely representative of their experience given the limitations of the data and potential approaches. It should also be noted that CPRD GP data does not include variables related to dementia severity, or stage of dementia at diagnosis. Severity and stage of dementia are important to identifying healthcare need, and understanding healthcare use. The changing nature of dementia need for people with dementia can change greatly in a short period of time, and so many people receive a later diagnosis – particularly from certain socio-demographic groups. We tried to minimise these issues but were limited in our approach. Future research should look to take our approaches and apply it to more complete/generalisable datasets. A long period of follow-up (up to 15 years after healthcare use trajectories), could mean people were lost from the data as they moved into long-term care moved GP, changed to a non-CPRD-registered GP, or withdrew consent for their data to be sent from their GP to CPRD. This could impact reliability and validity of mortality risk estimates. In this study, associations between membership of healthcare use clusters and risk of mortality were tested. However, regression analyses alone cannot clarify the direction of causality in these associations-based analyses [83]. With the association between differential healthcare use and mortality, it is important to note the potential importance of dementia severity (12), and healthcare need [84]. However, no dementia severity data was available in this study. Though the importance of healthcare need and comorbidity as factors in health outcomes have been discussed, it should be addressed in future research and would improve the efficiency and strength of future association-based findings.

Formal healthcare is one part of the care picture for PLWD. The majority of people receiving home-care services, and living in care homes have dementia [85], emphasising the important role social care services play in the care of PLWD. No social care use data was available for this study, but future research should endeavour to include temporal patterns in social care contact and care transitions in care to understand the collective impact overall service use can have on health outcomes in dementia. A further limitation of this study is the smaller membership of some healthcare use trajectory clusters. Of the eight clusters across both early- and late-onset populations, three clusters represented less than 10% of their respective overall population. This may limit the representativeness of these clusters of the general healthcare pathways of PLWD. PLWD who are more in the minority in their temporal use of healthcare services, also need their experience to be represented as well as those larger healthcare use clusters.

## Conclusion

This study has identified different trajectories in healthcare use among PLWD, how they relate to social and spatial inequalities, and the risk of subsequent mortality. Our findings point towards thinking beyond singular pathways for healthcare design at the population level to leverage the heterogeneity in experiences, as well the importance of identifying particular trajectories early before they become problematic. The benefits of person-centred care in dementia have been established for both PLWD and the wider health social care system [86]. Involving PLWD and informal carers in care discussions and decisions can help to better meet their needs. Our trajectories can help clinicians and others involved in care discussions to understand not only the current picture for a PLWD, but also what the future possibilities of their care could look like. It is a priority to make services more appropriate and accessible to the breadth of PLWD in need, and to promote better care quality for all PLWD. Future research should provide a more complete picture of care among PLWD, incorporating trajectories in health and social care use, and exploiting the complexity in different experiences and outcomes related to pathways through the health system.

### Supplementary Information

**Additional file 1: Appendix 1.** Loss to follow-up for early- and late-onset population, for 10 years after date of diagnosis.

**Additional file 2: Appendix 2.** Bayesian Information Criterion (BIC) and Log-likelihood (logLik) values for group-based trajectory models of one to ten groups (k) for both early- and late-onset sample populations.

**Additional file 3: Appendix 3.** Inclusion in GBTM analyses, missing data and those who died in early- and late-onset dementia populations, by explanatory factors.

**Additional file 4: Appendix 4.** Multinomial logistic regression output for likelihood of cluster membership based on socio-economic and geographic explanatory factors.

**Additional file 5: Appendix 5.** Cox Proportional Hazards regression outputs for association between mortality risk and explanatory factors.

**Additional file 6: Appendix 6.** Dementia Read codes for extraction of sample population, CPRD data.

**Additional file 7: Appendix 7.** Flowchart for sample selection and criteria for loss to follow-up for stratified early- and late-onset population.

## Data Availability

The data that support the findings of this study are provided by Clinical Practice Research Datalink (CPRD) but restrictions apply to the availability of these data, which were used under license for the current study, and so are not publicly available. Section 5 of the DSA states,*’The Customer shall not permit any third party in whole or in part to access, study, analyse, refer to or otherwise use the CPRD Data’.* As such the Data Sharing Agreement (DSA) between CPRD – on behalf of The Secretary of State for Health and Social Care—and the University of Liverpool the data on which the analyses in this research paper are based, is intended for the strict use by the parties names in the DSA, and as such data cannot be made publicly available. Please contact the corresponding author if you wish to discuss the data, or contact CPRD directly to discuss data access.

## Abbreviations

A&E: Accident & Emergency
ARC NWC: Applied Research Collaboration North West Coast
BIC: Bayesian Information Criterion
EHR: Electronic Health Records
ESPRC: Engineering and Physical Research Council
GBTM: Group-Based Trajectory Model
GP: General Practice
HR: Hazard Ratio
logLik: Log-Likelihood
NIHR: National Institute for Health Research
PLWD: People Living With Dementia
RR: Relative Risk
UKRI: United Kingdom Research Institute
