# Supplementary material for: Identifying longitudinal healthcare pathways and subsequent mortality for people living with dementia in England: an observational group-based trajectory analysis
Source: BMC Geriatr. 2024 Feb 14;24:150. doi: 10.1186/s12877-024-04744-5 (PMC10865521; doi:10.1186/s12877-024-04744-5)
Supplement: Supplementary file 1 — Additional file 1: Appendix 1. Loss to follow-up for early- and late-onset population, for 10 years after date of diagnosis. [file 12877_2024_4744_MOESM1_ESM.docx]

*Appendix 1: Loss to follow-up for early- and late-onset population, for 10 years after date of diagnosis*

| Year After Diagnosis | Early-Onset | | Late-Onset | |
| --- | --- | --- | --- | --- |
|  | Remaining | | Remaining | |
|  | (n) | % | (n) | % |
| Total | 5210 |  | 137077 |  |
| 0 | 5157 | 99.0% | 131749 | 96.1% |
| 1 | 4996 | 95.9% | 118836 | 86.7% |
| 2 | 4717 | 90.5% | 101273 | 73.9% |
| 3 | 4263 | 81.8% | 80948 | 59.0% |
| ***4*** | ***3732*** | ***71.6%*** | ***62264*** | ***45.4%*** |
| 5 | 3184 | 61.1% | 45943 | 33.5% |
| 6 | 2604 | 50.0% | 32843 | 24.0% |
| 7 | 2087 | 40.1% | 22598 | 16.5% |
| 8 | 1644 | 31.6% | 15289 | 11.2% |
| 9 | 1227 | 23.6% | 10171 | 7.4% |
| 10 | 923 | 17.7% | 6588 | 4.8% |
