# Supplementary material for: Identifying longitudinal healthcare pathways and subsequent mortality for people living with dementia in England: an observational group-based trajectory analysis
Source: BMC Geriatr. 2024 Feb 14;24:150. doi: 10.1186/s12877-024-04744-5 (PMC10865521; doi:10.1186/s12877-024-04744-5)
Supplement: Supplementary file 2 — Additional file 2: Appendix 2. Bayesian Information Criterion (BIC) and Log-likelihood (logLik) values for group-based trajectory models of one to ten groups (k) for both early- and late-onset sample populations. [file 12877_2024_4744_MOESM2_ESM.docx]

*Appendix 2: Bayesian Information Criterion (BIC) and Log-likelihood (logLik) values for group-based trajectory models of one to ten groups (k) for both early- and late-onset sample populations*

| No. # groups in model | Early-Onset | | Late-Onset | |
| --- | --- | --- | --- | --- |
|  | BIC | logLik | BIC | logLik |
| 1 | 167410.7 | -83597.2 | 281555.2 | -140663.8 |
| 2 | 162357.8 | -80957.6 | 273089.7 | -136317.3 |
| 3 | 155647.7 | -77494.4 | 261287.5 | -130302.4 |
| ***4*** | ***149099.4*** | ***-74102.3*** | ***258323.7*** | ***-128691.1*** |
| 5 | 14613.5 | -6883.9 | 12360.0 | -5605.8 |
| 6 | 5153.1 | -1932.4 | 24266.5 | -11564.2 |
| 7 | 5056.5 | -1775.9 | 8552.2 | -3458.8 |
| 8 | 4274.6 | -1276.8 | 8183.1 | -3165.6 |
| 9 | 3704.3 | -878.6 | 15753.1 | -6971.3 |
| 10 | -55039.0 | 28596.3 | 19281.3 | -8502.6 |
