# Supplementary material for: Identifying longitudinal healthcare pathways and subsequent mortality for people living with dementia in England: an observational group-based trajectory analysis
Source: BMC Geriatr. 2024 Feb 14;24:150. doi: 10.1186/s12877-024-04744-5 (PMC10865521; doi:10.1186/s12877-024-04744-5)
Supplement: Supplementary file 3 — Additional file 3: Appendix 3. Inclusion in GBTM analyses, missing data and those who died in early- and late-onset dementia populations, by explanatory factors. [file 12877_2024_4744_MOESM3_ESM.docx]

*Appendix 3: Inclusion in GBTM analyses, missing data and those who died in early- and late-onset dementia populations, by explanatory factors^[[1]](#footnote-1)^*

| Explanatory Factor | Of entire early-onset population | | | | Of entire late-onset population | | | |
| --- | --- | --- | --- | --- | --- | --- | --- | --- |
|  | GBTM-included | | Population with missing data | | GBTM-included | | % missing data | |
|  | # | % | % <5 years data | *% died* | # | % | % <5 years data | *% died* |
| Female | 2027 | 54.3% | 49.8% | 48.2% | 42638 | 68.9% | 65.0% | 64.2% |
| Male | 1705 | 45.7% | 50.2% | 51.8% | 19606 | 31.1% | 35.0% | 35.8% |
| Under45 | 77 | 2.1% | 1.8% | 1.5% |  | | | |
| 45-54 | 594 | 15.9% | 18.7% | 17.8% |  |  |  |  |
| 55-64 | 3061 | 82.0% | 79.6% | 80.7% |  |  |  |  |
| 65-74 |  | | | | 13343 | 21.1% | 9.6% | 7.4% |
| 75-84 |  |  |  |  | 32876 | 53.1% | 40.5% | 37.9% |
| 85-94 |  |  |  |  | 15521 | 25.0% | 44.8% | 48.3% |
| 95+ |  |  |  |  | 504 | 0.8% | 5.1% | 6.4% |
| Asian | 95 | 2.5% | 2.3% | 2.2% | 946 | 1.3% | 1.5% | 1.2% |
| Black | 88 | 2.4% | 3.0% | 2.3% | 1192 | 1.9% | 1.8% | 1.1% |
| Mixed/Other | 40 | 1.1% | 1.0% | 1.1% | 521 | 0.9% | 0.8% | 0.6% |
| White | 3267 | 87.5% | 87.2% | 94.4% | 56756 | 91.2% | 87.0% | 97.1% |
| Quintile 1 (Most deprived) | 724 | 19.4% | 20.0% | 21.8% | 9921 | 15.4% | 15.2% | 15.9% |
| Quintile 2 | 703 | 18.8% | 20.2% | 21.4% | 10793 | 18.1% | 17.5% | 17.5% |
| Quintile 3 | 771 | 20.7% | 20.4% | 19.4% | 12421 | 19.3% | 20.2% | 20.5% |
| Quintile 4 | 837 | 22.4% | 20.2% | 20.1% | 14297 | 23.0% | 23.1% | 23.4% |
| Quintile 5 (Least deprived) | 683 | 18.3% | 18.4% | 17.4% | 14707 | 24.0% | 23.6% | 22.7% |
| Rural | 498 | 13.3% | 12.5% | 12.1% | 8946 | 14.2% | 14.8% | 14.4% |
| Urban | 3234 | 86.7% | 87.5% | 87.9% | 53298 | 85.8% | 85.2% | 85.6% |
| East Midlands | 110 | 2.9% | 2.0% | 1.8% | 1307 | 2.3% | 2.1% | 1.9% |
| East of England | 189 | 5.1% | 5.1% | 3.5% | 3489 | 5.4% | 6.0% | 5.9% |
| London | 453 | 12.1% | 11.4% | 11.1% | 7032 | 10.7% | 9.6% | 8.9% |
| North East | 189 | 5.1% | 4.9% | 6.2% | 3471 | 5.6% | 4.9% | 5.3% |
| North West | 763 | 20.4% | 17.9% | 18.5% | 11396 | 18.9% | 17.4% | 17.9% |
| South Central | 516 | 13.8% | 13.3% | 14.6% | 8352 | 13.5% | 14.0% | 14.7% |
| South East Coast | 294 | 7.9% | 9.4% | 10.2% | 5061 | 8.4% | 8.8% | 8.5% |
| South West | 447 | 12.0% | 13.4% | 14.9% | 8907 | 14.2% | 15.0% | 15.2% |
| West Midlands | 617 | 16.5% | 17.9% | 15.4% | 10485 | 16.7% | 17.9% | 17.6% |
| Yorkshire & The Humber | 154 | 4.1% | 4.7% | 3.7% | 2744 | 4.3% | 4.2% | 4.0% |

1. *There are some members of early- and late-onset sample population who do not have Ethnicity or IMD 2015 deprivation quintile available in CPRD data, as such the sum total for such categories may be lower* [↑](#footnote-ref-1)
