# Supplementary material for: Identifying longitudinal healthcare pathways and subsequent mortality for people living with dementia in England: an observational group-based trajectory analysis
Source: BMC Geriatr. 2024 Feb 14;24:150. doi: 10.1186/s12877-024-04744-5 (PMC10865521; doi:10.1186/s12877-024-04744-5)
Supplement: Supplementary file 4 — Additional file 4: Appendix 4. Multinomial logistic regression output for likelihood of cluster membership based on socio-economic and geographic explanatory factors. [file 12877_2024_4744_MOESM4_ESM.docx]

*Appendix 4: Multinomial logistic regression output for likelihood of cluster membership based on socio-economic and geographic explanatory factors*

| Explanatory Factor | Early-Onset Dementia | | | | | | Late-Onset Dementia | | | | | |
| --- | --- | --- | --- | --- | --- | --- | --- | --- | --- | --- | --- | --- |
|  | Healthcare Trajectory Cluster (ref: cluster 3) | | | | | | Healthcare Trajectory Cluster (ref: cluster 4) | | | | | |
|  | Cluster 1 | | Cluster 2 | | Cluster 4 | | Cluster 1 | | Cluster 2 | | Cluster 3 | |
|  | Coef | Std.Er | Coef | Std.Er | Coef | Std.Er | Coef | Std.Er | Coef | Std.Er | Coef | Std.Er |
| ***(Intercept)*** | ***-2.559*** | ***0.451*** | ***-4.457*** | ***0.785*** | ***-0.215*** | ***0.187*** | ***-1.758*** | ***0.232*** | ***-0.517*** | ***0.152*** | ***-3.436*** | ***0.411*** |
| *Sex (ref: Female)* | | | | | | | | | | | | |
| Male | 0.205 | *0.158* | -0.342 | *0.199* | -0.050 | *0.073* | -0.042 | *0.098* | 0.046 | *0.062* | -0.142 | *0.125* |
| *Age Group (ref: 55-64)* | | | | | | | | | | | | |
| <45 | -0.066 | *0.611* | 1.048 | *0.462* | 0.198 | *0.261* |  | | | | | |
| 45-54 | -0.019 | *0.214* | -0.276 | *0.287* | -0.089 | *0.099* |  |  |  |  |  |  |
| 75-84 |  | | | | | | 0.130 | *0.114* | 0.153 | *0.074* | 0.330 | *0.159* |
| 85-94 |  |  |  |  |  |  | 0.043 | *0.138* | 0.438 | *0.086* | 0.853 | *0.170* |
| 95+ |  |  |  |  |  |  | -0.580 | *0.756* | 0.562 | *0.331* | 0.815 | *0.575* |
| *Ethnicity (ref: White)* | | | | | | | | | | | | |
| Asian | -0.785 | *0.735* | -11.999 | *187.462* | 0.155 | *0.220* | -0.879 | *0.534* | 0.170 | *0.241* | -0.606 | *0.611* |
| Black | 0.048 | *0.560* | -0.351 | *0.569* | -0.068 | *0.251* | -0.863 | *0.389* | -0.274 | *0.210* | -0.959 | *0.531* |
| Mixed/Other | 0.661 | *0.559* | -0.108 | *0.759* | -0.542 | *0.395* | -0.488 | *0.627* | 0.536 | *0.301* | -0.044 | *0.630* |
| *IMD 2015 Quintile (ref: Quintile 5: Least Deprived)* | | | | | | | | | | | | |
| Quintile 4 | -0.046 | *0.238* | 0.552 | *0.334* | 0.110 | *0.115* | 0.171 | *0.136* | 0.094 | *0.085* | 0.016 | *0.160* |
| Quintile 3 | -0.184 | *0.257* | 0.599 | *0.346* | 0.098 | *0.119* | -0.043 | *0.147* | -0.153 | *0.090* | -0.211 | *0.172* |
| Quintile 2 | -0.025 | *0.257* | 0.716 | *0.352* | 0.115 | *0.123* | 0.269 | *0.147* | 0.095 | *0.093* | 0.016 | *0.178* |
| Quintile 1 (Most deprived) | -0.195 | *0.272* | 0.828 | *0.368* | 0.033 | *0.127* | 0.489 | *0.152* | 0.050 | *0.101* | 0.124 | *0.189* |
| *Urban-Rural GP Classification (ref: Urban)* | | | | | | | | | | | | |
| Rural | 0.153 | *0.240* | 0.493 | *0.283* | 0.217 | *0.111* | -0.094 | *0.143* | 0.105 | *0.085* | 0.129 | *0.164* |
| *GP Region (ref: North East)* | | | | | | | | | | | | |
| North West | 0.361 | *0.427* | 0.923 | *0.756* | -0.219 | *0.175* | 0.069 | *0.207* | 0.221 | *0.137* | 1.262 | *0.384* |
| Yorkshire & The Humber | 0.071 | *0.559* | 0.820 | *0.884* | -0.324 | *0.235* | -0.150 | *0.294* | 0.101 | *0.184* | 1.119 | *0.448* |
| East Midlands | 0.251 | *0.620* | 1.249 | *0.941* | 0.060 | *0.263* | -0.330 | *0.421* | 0.348 | *0.224* | 1.326 | *0.504* |
| East of England | -0.307 | *0.585* | 1.005 | *0.862* | -0.336 | *0.228* | 0.109 | *0.271* | 0.034 | *0.176* | 0.882 | *0.451* |
| West Midlands | 0.092 | *0.448* | 0.744 | *0.779* | -0.184 | *0.180* | 0.026 | *0.216* | 0.310 | *0.140* | 1.212 | *0.390* |
| London | -0.043 | *0.483* | 1.763 | *0.761* | -0.371 | *0.197* | 0.238 | *0.229* | 0.176 | *0.153* | 1.440 | *0.400* |
| South East Coast | 0.101 | *0.494* | 1.653 | *0.781* | -0.405 | *0.210* | 0.211 | *0.245* | 0.200 | *0.161* | 1.431 | *0.409* |
| South Central | 0.476 | *0.453* | 1.766 | *0.767* | 0.016 | *0.190* | 0.313 | *0.220* | 0.079 | *0.148* | 1.177 | *0.399* |
| South West | 0.170 | *0.460* | 1.040 | *0.780* | -0.225 | *0.189* | 0.122 | *0.214* | -0.023 | *0.144* | 0.895 | *0.397* |
