# Supplementary material for: Identifying longitudinal healthcare pathways and subsequent mortality for people living with dementia in England: an observational group-based trajectory analysis
Source: BMC Geriatr. 2024 Feb 14;24:150. doi: 10.1186/s12877-024-04744-5 (PMC10865521; doi:10.1186/s12877-024-04744-5)
Supplement: Supplementary file 5 — Additional file 5: Appendix 5. Cox Proportional Hazards regression outputs for association between mortality risk and explanatory factors. [file 12877_2024_4744_MOESM5_ESM.docx]

*Appendix 5: Cox Proportional Hazards regression outputs for association between mortality risk and explanatory factors^[[1]](#footnote-1)^*

| **Explanatory Factor** | **Early-onset dementia** | | | | **Late-onset dementia** | | | |
| --- | --- | --- | --- | --- | --- | --- | --- | --- |
|  | **Hazard Ratio (HR)** | ***95% Confidence Intervals*** | **p-value** | **sig** | **Hazard Ratio (HR)** | ***95% Confidence Intervals*** | **p-value** | **sig** |
| Cluster 1 | 2.21 | *(1.78 – 2.75)* | 0.00 | *** | 1.08 | *(0.96 – 1.21)* | 0.21 |  |
| Cluster 2 | 0.47 | *(0.28 – 0.77)* | 0.00 | ** | 0.72 | *(0.66 – 0.80)* | 0.00 | *** |
| Cluster 3 | *Not Applicable (reference group for GBTM)* | | | | 0.32 | *(0.25 – 0.40)* | 0.00 | *** |
| Cluster 4 | 1.37 | *(1.21 – 1.56)* | 0.00 | *** | *Not Applicable (reference group for GBTM)* | | | |
| Age At Diagnosis | 1.02 | *(1.00 – 1.03)* | 0.01 | * | 1.06 | *(1.05 – 1.07)* | 0.00 | *** |
| Male | 1.09 | *(0.97 – 1.23)* | 0.14 |  | 1.21 | *(1.11 – 1.32)* | 0.00 | *** |
| Asian | 0.72 | *(0.45 – 1.13)* | 0.15 |  | 1.03 | *(0.71 – 1.48)* | 0.89 |  |
| Black | 0.98 | *(0.64 – 1.49)* | 0.92 |  | 0.89 | *(0.65 – 1.21)* | 0.46 |  |
| Mixed/Other | 0.81 | *(0.42 – 1.58)* | 0.54 |  | 1.33 | *(0.87 – 2.03)* | 0.19 |  |
| Quintile 4 | 1.04 | *(0.86 – 1.26)* | 0.66 |  | 1.06 | *(0.94 – 1.19)* | 0.36 |  |
| Quintile 3 | 1.13 | *(0.93 – 1.38)* | 0.23 |  | 1.05 | *(0.93 – 1.19)* | 0.45 |  |
| Quintile 2 | 1.16 | *(0.95 – 1.42)* | 0.15 |  | 1.05 | *(0.92 – 1.20)* | 0.46 |  |
| Quintile 1 (Most Deprived) | 1.02 | *(0.82 – 1.26)* | 0.86 |  | 1.16 | *(1.01 – 1.33)* | 0.03 | * |
| Rural | 1.00 | *(0.83 – 1.20)* | 0.99 |  | 1.01 | *(0.90 – 1.14)* | 0.87 |  |
| North West | 1.04 | *(0.77 – 1.40)* | 0.81 |  | 0.88 | *(0.73 – 1.05)* | 0.15 |  |
| Yorkshire & The Humber | 0.99 | *(0.67 – 1.49)* | 0.98 |  | 0.82 | *(0.64 – 1.05)* | 0.11 |  |
| East Midlands | 0.78 | *(0.49 – 1.24)* | 0.29 |  | 0.95 | *(0.70 – 1.30)* | 0.75 |  |
| East of England | 1.27 | *(0.87 – 1.85)* | 0.22 |  | 0.81 | *(0.64 – 1.02)* | 0.07 |  |
| West Midlands | 0.95 | *(0.69 – 1.29)* | 0.73 |  | 0.78 | *(0.65 – 0.94)* | 0.01 | ** |
| London | 0.94 | *(0.67 – 1.32)* | 0.72 |  | 0.63 | *(0.51 – 0.78)* | 0.00 | *** |
| South East Coast | 1.07 | *(0.75 – 1.53)* | 0.71 |  | 0.64 | *(0.51 – 0.80)* | 0.00 | *** |
| South Central | 1.24 | *(0.91 – 1.70)* | 0.17 |  | 1.01 | *(0.83 – 1.22)* | 0.93 |  |
| South West | 1.10 | *(0.80 – 1.51)* | 0.58 |  | 0.91 | *(0.75 – 1.09)* | 0.30 |  |
| *Please note significance levels: '***' = 0; '**' = 0.001; '*' = 0.01 ‘*’’ = 0.05* | | | | | | | | |

1. *Reference groups for explanatory factors: Healthcare cluster: Early-onset = cluster 3; Late-onset = cluster 4; Sex = female; Ethnicity = White; IMD 2015 Deprivation Quintile = Quintile 5 (Least Deprived); Urban-Rural GP classification = Urban; GP Region = North East; as a continuous variable there is no reference for Age At Diagnosis* [↑](#footnote-ref-1)
