# Supplementary material for: Identifying longitudinal healthcare pathways and subsequent mortality for people living with dementia in England: an observational group-based trajectory analysis
Source: BMC Geriatr. 2024 Feb 14;24:150. doi: 10.1186/s12877-024-04744-5 (PMC10865521; doi:10.1186/s12877-024-04744-5)
Supplement: Supplementary file 6 — Additional file 6: Appendix 6. Dementia Read codes for extraction of sample population, CPRD data. [file 12877_2024_4744_MOESM6_ESM.docx]

**Appendix 6: Dementia Read codes for extraction of sample population, CPRD data**

| code | coding_system | description |
| --- | --- | --- |
| A411.00 | Read | Jakob-Creutzfeldt disease |
| E00..00 | Read | Senile and presenile organic psychotic conditions |
| E000.00 | Read | Uncomplicated senile dementia |
| E001.00 | Read | Presenile dementia |
| E001000 | Read | Uncomplicated presenile dementia |
| E00..11 | Read | Senile dementia |
| E001100 | Read | Presenile dementia with delirium |
| E00..12 | Read | Senile/presenile dementia |
| E001200 | Read | Presenile dementia with paranoia |
| E001300 | Read | Presenile dementia with depression |
| E001z00 | Read | Presenile dementia NOS |
| E002.00 | Read | Senile dementia with depressive or paranoid features |
| E002000 | Read | Senile dementia with paranoia |
| E002100 | Read | Senile dementia with depression |
| E002z00 | Read | Senile dementia with depressive or paranoid features NOS |
| E003.00 | Read | Senile dementia with delirium |
| E004.00 | Read | Arteriosclerotic dementia |
| E004000 | Read | Uncomplicated arteriosclerotic dementia |
| E004100 | Read | Arteriosclerotic dementia with delirium |
| E004.11 | Read | Multi infarct dementia |
| E004200 | Read | Arteriosclerotic dementia with paranoia |
| E004300 | Read | Arteriosclerotic dementia with depression |
| E004z00 | Read | Arteriosclerotic dementia NOS |
| E00y.00 | Read | Other senile and presenile organic psychoses |
| E00y.11 | Read | Presbyophrenic psychosis |
| E00z.00 | Read | Senile or presenile psychoses NOS |
| E012.00 | Read | Other alcoholic dementia |
| E012000 | Read | Chronic alcoholic brain syndrome |
| E012.11 | Read | Alcoholic dementia NOS |
| E02y100 | Read | Drug-induced dementia |
| E041.00 | Read | Dementia in conditions EC |
| Eu00.00 | Read | [X]Dementia in Alzheimer's disease |
| Eu00000 | Read | [X]Dementia in Alzheimer's disease with early onset |
| Eu00011 | Read | [X]Presenile dementia;Alzheimer's type |
| Eu00012 | Read | [X]Primary degen dementia; Alzheimer's type; presenile onset |
| Eu00013 | Read | [X]Alzheimer's disease type 2 |
| Eu00100 | Read | [X]Dementia in Alzheimer's disease with late onset |
| Eu00111 | Read | [X]Alzheimer's disease type 1 |
| Eu00112 | Read | [X]Senile dementia;Alzheimer's type |
| Eu00113 | Read | [X]Primary degen dementia of Alzheimer's type; senile onset |
| Eu00200 | Read | [X]Dementia in Alzheimer's dis; atypical or mixed type |
| Eu00z00 | Read | [X]Dementia in Alzheimer's disease; unspecified |
| Eu00z11 | Read | [X]Alzheimer's dementia unspec |
| Eu01.00 | Read | [X]Vascular dementia |
| Eu01000 | Read | [X]Vascular dementia of acute onset |
| Eu01100 | Read | [X]Multi-infarct dementia |
| Eu01.11 | Read | [X]Arteriosclerotic dementia |
| Eu01111 | Read | [X]Predominantly cortical dementia |
| Eu01200 | Read | [X]Subcortical vascular dementia |
| Eu01300 | Read | [X]Mixed cortical and subcortical vascular dementia |
| Eu01y00 | Read | [X]Other vascular dementia |
| Eu01z00 | Read | [X]Vascular dementia; unspecified |
| Eu02.00 | Read | [X]Dementia in other diseases classified elsewhere |
| Eu02000 | Read | [X]Dementia in Pick's disease |
| Eu02100 | Read | [X]Dementia in Creutzfeldt-Jakob disease |
| Eu02200 | Read | [X]Dementia in Huntington's disease |
| Eu02300 | Read | [X]Dementia in Parkinson's disease |
| Eu02400 | Read | [X]Dementia in human immunodef virus [HIV] disease |
| Eu02500 | Read | [X]Lewy body dementia |
| Eu02y00 | Read | [X]Dementia in other specified diseases classif elsewhere |
| Eu02z00 | Read | [X] Unspecified dementia |
| Eu02z11 | Read | [X] Presenile dementia NOS |
| Eu02z12 | Read | [X] Presenile psychosis NOS |
| Eu02z13 | Read | [X] Primary degenerative dementia NOS |
| Eu02z14 | Read | [X] Senile dementia NOS |
| Eu02z15 | Read | [X] Senile psychosis NOS |
| Eu02z16 | Read | [X] Senile dementia; depressed or paranoid type |
| Eu04100 | Read | [X]Delirium superimposed on dementia |
| Eu05700 | Read | [X]Mild cognitive disorder |
| F110.00 | Read | Alzheimer's disease |
| F110000 | Read | Alzheimer's disease with early onset |
| F110100 | Read | Alzheimer's disease with late onset |
| F111.00 | Read | Pick's disease |
| F1110A | Read |  |
| F112.00 | Read | Senile degeneration of brain |
| F116.00 | Read | Lewy body disease |
| F118.00 | Read |  |
| F11x.00 | Read | Cerebral degeneration in other disease EC |
| F11x000 | Read | Cerebral degeneration due to alcoholism |
| F11x011 | Read | Alcoholic encephalopathy |
| F11x200 | Read | Cerebral degeneration due to cerebrovascular disease |
| F11x400 | Read | Cerebral degeneration due to neoplastic disease |
| F11x500 | Read | Cerebral degeneration due to myxoedema |
| F11x600 | Read | Cerebral degeneration due to vitamin B12 deficiency |
| F11x700 | Read | Cerebral degeneration due to Jakob - Creutzfeldt disease |
| F11x800 | Read | Cerebral degeneration due to multifocal leucoencephalopathy |
| F11x900 | Read | Cerebral degeneration in Parkinson's disease |
| F11xz00 | Read | Cerebral degeneration other disease NOS |
| F11y.00 | Read | Other cerebral degeneration |
| F11y000 | Read | Reye's syndrome |
| F11y100 | Read | Cerebral ataxia |
| F11yz00 | Read | Other cerebral degeneration NOS |
| F11z.00 | Read | Cerebral degeneration NOS |
| F11z.11 | Read | Cerebral atrophy |
| F134.00 | Read | Huntington's chorea |
| Fyu3000 | Read | [X]Other Alzheimer's disease |
