# Supplementary material for: Identifying longitudinal healthcare pathways and subsequent mortality for people living with dementia in England: an observational group-based trajectory analysis
Source: BMC Geriatr. 2024 Feb 14;24:150. doi: 10.1186/s12877-024-04744-5 (PMC10865521; doi:10.1186/s12877-024-04744-5)
Supplement: Supplementary file 7 — Additional file 7: Appendix 7. Flowchart for sample selection and criteria for loss to follow-up for stratified early- and late-onset population. [file 12877_2024_4744_MOESM7_ESM.docx]

**Appendix 7: Flowchart for sample selection and criteria for loss to follow-up for stratified early- and late-onset population**

| Included | n | Excluded |  | Included | n | Excluded |
| --- | --- | --- | --- | --- | --- | --- |
| Total initial early-onset population | 5210 |  |  | Total initial early-onset population | 137077 |  |
|  | *1475* | *<5 years post-diagnosis healthcare use data* |  |  | *74813* | *<5 years post-diagnosis healthcare use data* |
| Total available for GBTM-inclusion | 3735 |  |  | Total available for GBTM-inclusion | 62264 |  |
|  | *3* | *Incomplete socio-demographic data* |  |  | *24* | *Incomplete socio-demographic data* |
| Total included in GBTM | 3732 |  |  | 10% total included in GBTM | 6224 |  |
|  | *1126* | *Loss to follow-up due to mortality during study* |  |  | *2548* | *Loss to follow-up due to mortality during study* |
|  | *2595* | *Loss to follow-up (healthcare data incomplete)* |  |  | *3674* | *Loss to follow-up (healthcare data incomplete)* |
| Complete data until study end | *11* |  |  | Complete data until study end | *2* |  |
